# Slow Blink Eye Closure in Shelter Cats Is Related to Quicker Adoption

**DOI:** 10.3390/ani10122256

**Published:** 2020-11-30

**Authors:** Tasmin Humphrey, Faye Stringer, Leanne Proops, Karen McComb

**Affiliations:** 1 Mammal Communication and Cognition Research Group, School of Psychology, University of Sussex, Brighton BN1 9QH, UK; fayestringer@gmail.com; 2 Centre for Comparative and Evolutionary Psychology, Department of Psychology, University of Portsmouth, Portsmouth PO1 2DY, UK; leanne.proops@port.ac.uk

**Keywords:** human-animal interactions, facial expressions, cats, slow blink

## Abstract

**Simple Summary:**

Slow blinking is a type of interaction between humans and cats that involves a sequence of prolonged eye narrowing movements being given by both parties. This interspecific social behaviour has recently been studied empirically and appears to be a form of positive communication for cats, who are more likely to approach a previously unfamiliar human after such interactions. We investigated whether slow blinking can also affect human preferences for cats in a shelter environment. We measured whether cats’ readiness to respond to a human-initiated slow blink interaction was associated with rates of rehoming in the shelter. We also examined cats’ propensity to slow blink when they were anxious around humans or not. We demonstrated that cats that responded to human slow blinking by using eye closures themselves were rehomed quicker than cats that closed their eyes less. Cats that were initially identified as more nervous around humans also showed a trend towards giving longer total slow blink movements in response to human slow blinking. Our results suggest that the cat slow blink sequence is perceived as positive by humans and may have a dual function in cats, occurring in both affiliative and submissive situations.

**Abstract:**

The process of domestication is likely to have led to the development of adaptive interspecific social abilities in animals. Such abilities are particularly interesting in less gregarious animals, such as cats. One notable social behaviour that cats exhibit in relation to humans is the slow blink sequence, which our previous research suggests can function as a form of positive communication between cats and humans. This behaviour involves the production of successive half blinks followed by either a prolonged narrowing of the eye or an eye closure. The present study investigates how cat (*n* = 18) slow blink sequences might affect human preferences during the adoption of shelter cats. Our study specifically tested (1) whether cats’ propensity to respond to human-initiated slow blinking was associated with their speed of rehoming from a shelter environment, and (2) whether cats’ anxiety around humans was related to their tendency to slow blink. Our experiments demonstrated that cats that showed an increased number of and longer eye closures in response to human slow blinks were rehomed faster, and that nervous cats, who had been identified as needing desensitisation to humans, tended to spend more time producing slow blink sequences in response to human slow blinks than a non-desensitisation group. Collectively, these results suggest that the cat slow blink sequence is perceived as positive by humans and may have a dual function—occurring in both affiliative and submissive contexts.

## 1. Introduction

Human attitudes towards animals can be described in terms of two primary dimensions—affect and utility [1]. The domestication of *Felis catus*, the cat, is thought to have been driven by their use as a means of pest control [2]. Thus, utility initially described early human motivations to tolerate a proximity to cats. However, over time the cat has integrated itself into the family home, becoming nearly as prevalent in UK households as the domestic dog [3]. Now, cats seem to play an increasingly significant affective role in our lives, often providing a source of emotional support to owners [4]. This shift from co-existence with humans to companion raises interesting questions regarding the particular social behaviours in cats necessary for the formation and maintenance of the cat–human bond.

Despite previously being solitary animals, cats have become facultatively social during the process of domestication. They have been shown to use human given cues [5,6,7] and adapt their own vocal communication to gain food and care in heterospecific interactions by using a solicitation purr, where a high frequency element embedded in the low frequency purr adds perceived urgency, apparently through its similarity to a human infant cry [8]. Social skills are advantageous to individuals [9], in part due to signalling motivation to others, for example via facial expression [10]. Facial expressions therefore serve specific functions in social contexts, for example expressing negative emotions such as fear can alert the receiver to an aversive situation. However, the scientific study of communication in animals during positive contexts remains relatively scant [11]. A proposed function of positive expressions in humans is to build on personal resources, including social relationships [12]. This constructive function may extend to animals as well, since more socially tolerant macaques, where interactions are less dictated by strict social structures, have a wider repertoire of affiliative facial displays [13]. In addition, the degree to which cats display affection has been shown to be associated with owners’ reported levels of attachment [14]. Thus, further investigation into cat–human positive communication could shed light on the social function of positive communication, specifically in the context of our relationship with felines.

**Figure 1:**
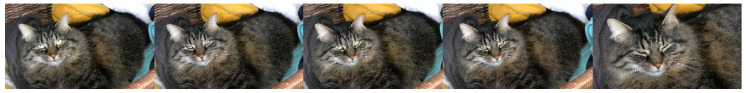
Still images captured of the cat slow blink sequence, starting from a neutral face followed by a half blink then eye narrowing moving towards eye closure.

One cat-human signal that has recently been documented scientifically is the slow blink sequence. Cat slow blink sequences involve narrowing of the eye aperture, specifically consisting of a series of shorter half blinks, followed by either a stable narrowing of the eye or a prolonged eye closure (see Figure 1). Cats appear to respond to similar eye narrowing movements initiated by humans, and tend to approach previously unfamiliar humans after such slow blink interactions [15]. The slow blink has also been noted when a cat is seeking reassurance in a tense environment [16]. A survey into feline behaviour by the animal welfare charity, Cats Protection, found that 69% of the 1100 cat owners asked indicated that the slow blink implies a relaxed cat [17].

Slow blinking in cats may have evolved in response to human preferences for positive-looking facial expressions, particularly because slow blinking in cats shares features with the human Duchenne smile (i.e., narrowing of the eyes). Humans are able to detect positive facial expressions using only upper facial cues [18], as well as indirectly through unfocused images [19], for a review see, [20]. Happy faces also lead to more positive inferences regarding others’ interpersonal traits such as kindness and affiliation [21,22]. To examine the functional relevance of specific behaviours produced by companion animals when interacting with humans, preference tests can be used. [23] found that dogs using a specific facial expression (Action Unit (AU) 101, the inner brow raiser) were preferred by humans in terms of the rate of rehoming in an animal shelter. In their study, adoption speed in a shelter environment was used as a proxy for selection of dogs over time, a measure that we will also use to explore human preference for adopting shelter cats.

In the current study, we specifically aimed to investigate how human-cat slow blinking interactions affect the speed of adoption of cats in a shelter environment. We tested whether shelter cats responded more to experimenter-initiated slow blink interactions compared to a control trial in which the experimenter adopted a neutral facial expression. We also examined whether cats’ responses were related to rehoming speed over time. Finally, we compared whether cats that had on admission been assessed as showing more anxiety around humans responded differently to slow blink interactions than those who were not deemed anxious. Cats’ eye narrowing movements were recorded using the Cat Facial Action Coding System (CatFACS) [24], an anatomically based system for coding facial muscle movements. We predicted that cats would be more responsive to the experimenter’s slow blinking, by also narrowing their eye aperture, compared to the neutral expression. We also predicted that cats that were more responsive to slow blinking would be rehomed sooner, and that propensity to slow blink would vary between anxious and non-anxious cats.

## 2. Materials and Methods

### 2.1. Subjects

Cats were recruited from Cats Protection at The National Cat Adoption Centre (NCAC) in Sussex. Data collection took place over 10 days between 27 June 2017 and 18 July 2017. Twenty-four cats in total were filmed. Selection of the cats was based on which cats were visible inside their pen (i.e., cats who were not in the outside area) and cats that were awake at the time of filming. Six cats were removed from the final analysis due to their lack of attentiveness to the researcher during the slow blinking interaction or lack of visibility when coding the videos. Of the 18 remaining cats, 9 were female and 9 were male. All cats were neutered and had no medical issues. Adult cats that were ≥1 year old were included in the study, and ages ranged from 1 to 16 years (Mean (M) = 6.62 ± 4.56 Standard Deviation (SD)).

Staff members on admission to the Cats Protection site observe cats to check for signs of anxiousness (e.g., hiding, reluctance to eat or drink). Anxious cats are placed in a desensitisation programme in which Cats Protection employees and volunteers spend extra time in contact with the cat to enhance the cat’s confidence around humans. The final sample included 8 cats in the desensitisation group and 10 in the non-desensitisation group.

### 2.2. Experimental Procedure

**Figure 2:**
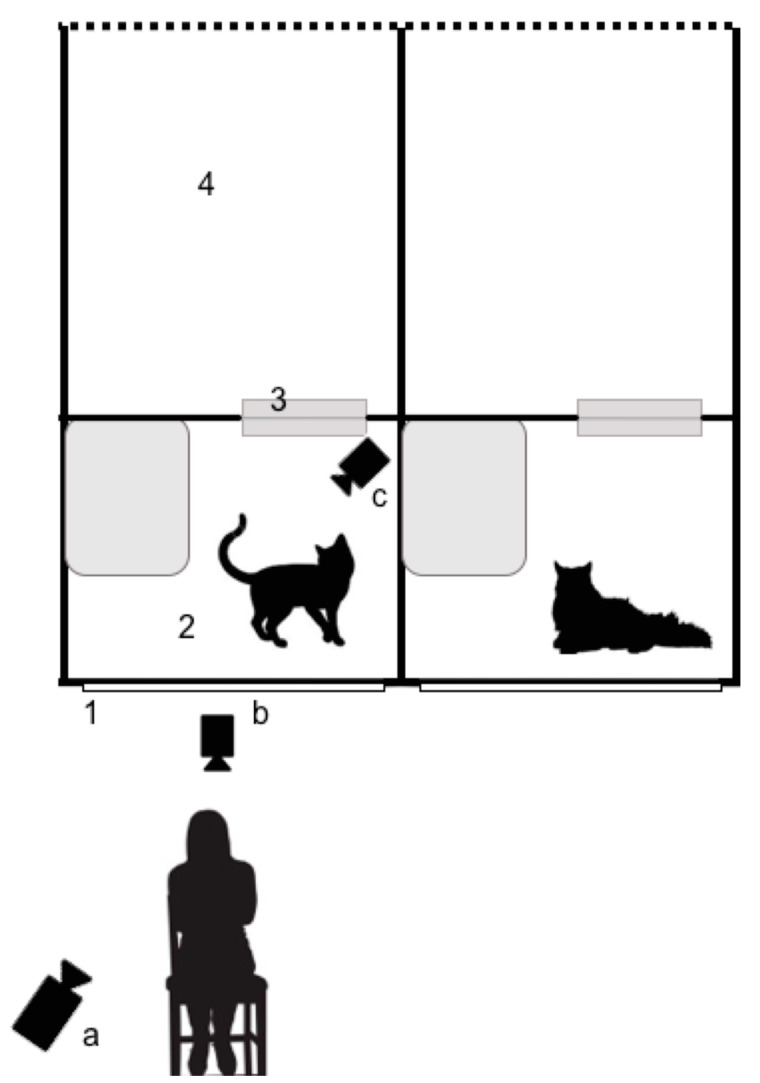
Experimental set-up (1 = pen door, 2 = indoor pen, 3 = cat flap, 4 = back enclosure; a = Panasonic HC-V270, b and c = GoPro HERO4).

Cats were housed in a homing wing of the NCAC, consisting of parallel rows of pens. The dimensions of the inside of each pen were 84 cm × 84 cm × 84 cm and contained an elevated area. Cats also had access to a larger, partially outdoor enclosure that was connected to the inside pen by a cat flap. They had visual access to an internal corridor via a glass screen door that was located at eye level when the experimenter was seated on a chair. Cats had enrichment toys and a bed in the pen at all times. Video footage of inside the cat’s pen was obtained using a Panasonic HC-V270 (Panasonic, Osaka, Japan) placed 60 cm away from the screen door. A GoPro HERO4 camera (Woodman Labs Inc., San Mateo, CA, USA) was also used to capture footage inside the pen to increase the likelihood of recording the cat’s eye movements. Another GoPro HERO4 camera was placed outside of the pen directly in front of the female experimenter (FS; see Figure 2). Once cameras were in place, cats were given 5 min to habituate to the presence of the equipment without the experimenter present. Each cat participated in four trials (2 experimental and 2 control), counterbalanced by condition. The first two trials were not included in the analyses but were used to allow the cats to habituate to the conditions.

In the slow blinking trial, the experimenter sat in front of the screen door and attempted to engage the cat in an interaction by slowly narrowing and closing her eyes towards the cat in order to initiate a slow blinking interaction. Where appropriate, the experimenter called the cat’s attention back to the interaction when the cat’s gaze diverted from the experimenter. The slow blink stimulus was then repeated several times throughout the trial. Control trials had the experimenter seated in the same position as the slow blink trials; however, the experimenter adopted a neutral expression and averted her gaze slightly to the left of the pen at human eye level whilst still facing the cat. The experimenter could blink as normal (<500 ms). This eye position was chosen as previous research has revealed that cats may perceive staring as threatening [15]. All trials lasted for 60 s and the inside camera was disinfected between testing different subjects using Anistel^®^ (Tristel, Cambridge, UK) for both infectious disease control and to remove possible effects of scent.

### 2.3. Behavioural and Statistical Analyses

Experimenter and cat eye narrowing movements in trials 3 and 4 were blind coded from videos on an Mini Mac computer (Apple Inc, Cupertino, CA, USA) using Sportscode Gamebreaker Plus^®^ 10.3 (Hudl, Lincoln, NE, USA) (www.hudl.com) software. Eye narrowing movements were derived from CatFACS [24] as well as adapted coding schemes used for slow blink research (see Table 1; [15]). Eye responses that may have occurred due to the experimenter calling the cat’s name to gain their attention were controlled for by excluding any cat eye movements made within half a second of an experimenter’s call in the absence of an experimenter eye closure. Coders were certified in CatFACS (TH and FS) and inter-rater reliability tests between TH and FS using identical codes found a Cronbach’s alpha of 0.9.

Data consisted of the number of instances and duration of individual eye movements (half blink, eye closure and eye narrowing). Cat’s individual eye movements were also summed to create a total cat eye movement score. A total response latency measure was calculated for each cat’s slow blinking trial by summing all of the latencies to the start of the cat’s eye movement that occurred within 10 s of the experimenter’s eye movement (note here that a larger score would indicate a slower total response latency). Slow blinks given by the experimenter that were either not responded to or responded to after 10 s by cats were assigned a latency of 10.1. All latencies and non-responses were then summed together and divided by the number of slow blinks delivered by the experimenter for each cat. Adoption rates were measured as days before the cat was reserved to be rehomed, with a maximum date of 132 days. Cats who were not adopted after 132 days were assigned a value of 133 days in the analyses (*n* = 4).

All analyses were conducted with SPSS Statistics 24 software (IBM, Chicago, IL, USA). Wilcoxon tests (Z) were used to examine differences in the cat’s specific eye narrowing movements (half blink, eye narrowing, and eye closure) between the slow blink stimulus and the neutral condition. Spearman’s rank correlations (r) assessed the relationship between the cat’s specific eye narrowing movements and days before cats were reserved for rehoming. Mann–Whitney U tests were calculated to compare the response latency scores and eye movements of anxious cats that had been selected for a desensitisation treatment at the shelter and cats that did not require desensitisation treatment. We also calculated the effect sizes (Cohen’s d) to interpret the results for the human-initiated slow blinking and for the comparison of desensitisation and non-desensitisation cats.

### 2.4. Ethical Statement

This study was conducted in accordance with the Association for the Study of Animal Behaviour (ASAB) guidelines for the use of animals in research and was approved by both the University of Sussex Animal Ethical Review Committee (ERC), reference number: Non-ASPA—Nov2013; and Cats Protection.

## 3. Results

### 3.1. Effects of Human-Initiated Slow Blinking

**Figure 3:**
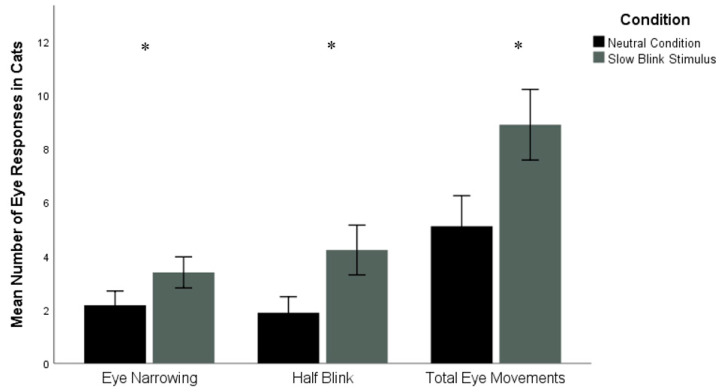
Mean number of instances of cat eye narrowing, half blinks, and total eye movements in the control versus slow blinking trials (*n* = 18). Error bars represent ±1 standard error. * *p* < 0.05.

The number of cat half blinks was significantly higher in the slow blinking trials (M = 4.22 ± 3.93 (SD)) compared to the control trials (M = 1.89 ± 2.52), Z = −2.01, *p* = 0.04, d = 0.71. There were also significantly more instances of eye narrowing in the slow blink stimulus condition (M = 3.39 ± 2.45) compared to the neutral condition (M = 2.17 ± 2.26), Z = −2.03, *p* = 0.04, d = 0.52. The number of total eye movements were significantly higher in the slow blink stimulus condition (M = 8.89 ± 5.58) compared to the neutral condition (M = 5.11 ± 4.81), Z = −2.31, *p* = 0.02, d = 0.73, (see Figure 3). No significant difference was found for the number of cat eye closures between slow blinking (M = 1.28 ± 1.64) and control trials (M = 1.06 ± 1.16), Z = −0.69, *p* = 0.49).

Tests also indicated that the duration of cat half blinks was significantly longer in the slow blinking trials (M = 2.69 ± 2.83) compared to the control trials (M = 1.06 ± 1.37, Z = −2.27, *p* = 0.02, d = 0.73). The duration of cat eye narrowing approached significance between the slow blinking condition (M = 10.58 ± 11.68) and the control condition (M = 8.42 ± 12.44), Z = −1.71, *p* = 0.09, d = 0.18. Finally, no significant differences were found between the slow blinking condition and the control condition in the durations of cat eye closure (slow blink: M = 10.90 ± 17.42; control: M = 14.72 ± 22.45) and total cat eye movements (slow blink: M = 24.17 ± 22.55; control: 24.19 ± 24.54), Z = −1.22, *p* = 0.22; Z = −0.07, *p* = 0.95, respectively.

### 3.2. Relationship between Eye Movements and Time to Rehome

**Figure 4:**
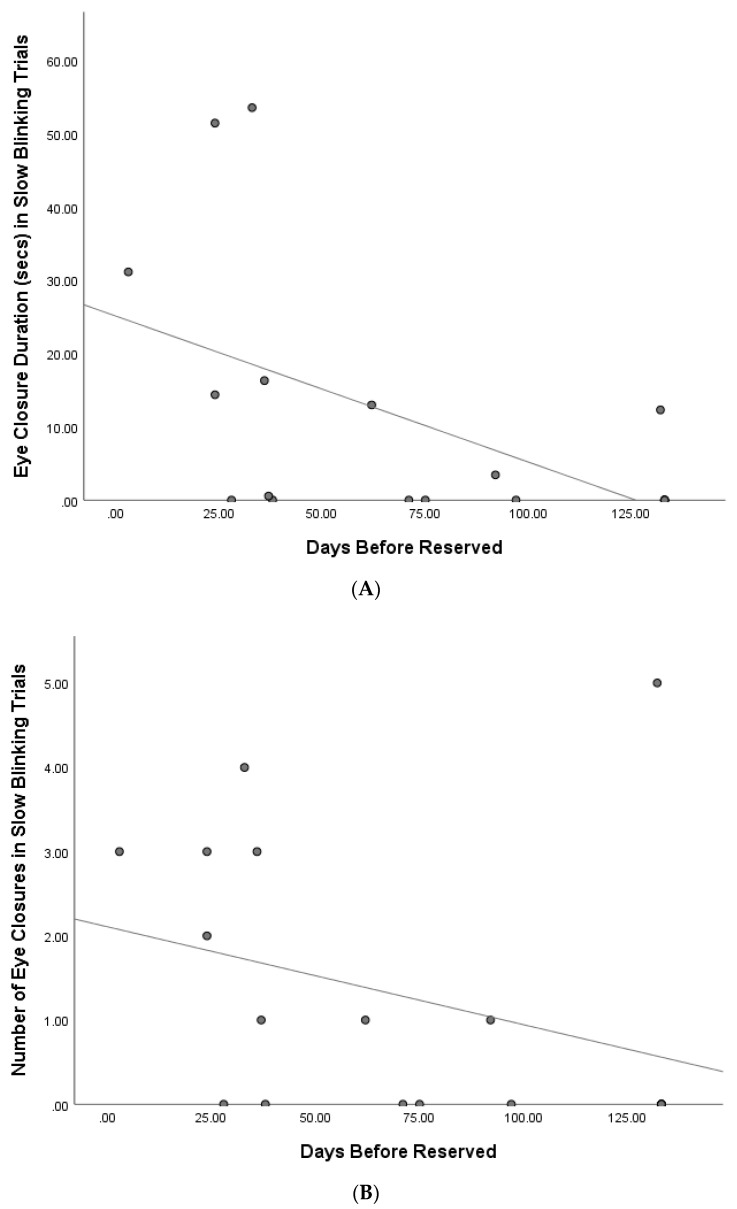
(**A**) Relationship between duration of cat eye closures and days before cats were reserved for rehoming; (**B**) relationship between the number of cat eye closures and days before cats were reserved for rehoming.

At the time of analysis, 14 cats (of *n* = 18) had been reserved to be rehomed. There was a significant negative correlation between the number and duration of cat eye closures in the slow blinking trials and the days before being reserved (frequency: r = −0.55, *p* = 0.02; duration: r = −0.67, *p* = 0.002; see Figure 4), and thus, as eye closure increased, cats took less time to rehome.

A Spearman’s correlation approached a significant relationship between duration of total eye movements in the slow blinking condition and days before cats were reserved (r = −0.45, *p* = 0.06). No significant correlations with days before being reserved were found for total response latency (r = −0.03, *p* = 0.92), or the durations and number of cat half blinks and eye narrowing, or number of total eye movements (half blink frequency: r = 0.26, *p* = 0.30; duration: r = 0.20, *p* = 0.42; eye narrowing frequency: r = −0.03, *p* = 0.89; duration: r = −0.31, *p* = 0.21; total eye movements frequency: r = 0.02, *p* = 0.94).

### 3.3. Comparison of Desensitisation and Non-Desensitisation Cats

The duration of total cat eye movements approached significance (U = 19.50, *p* = 0.07, d = 0.85), with the desensitisation group showing cumulatively longer total eye movements (M = 34.35 ± 24.22) than the non-desensitisation group (M = 16.03 ± 18.39). No significant difference was found between desensitisation and non-desensitisation groups in duration or number of half blinks, eye narrowing, and eye closure, or average response latency (see Appendix A for statistical results, and Table S1 for the full dataset).

## 4. Discussion

This study supports previous research that has shown that cats actively choose to engage in slow blinking with humans by responding with eye narrowing movements of their own. Our results offer additional insights into understanding how slow blinking functions in cat-human communication. Moreover, this study demonstrates for the first time that cats that responded to human slow blinking, specifically by using eye closures, were rehomed quicker than cats that closed their eyes less. This suggests that the use of slow blinking may have given cats a selective advantage during the domestication process. Furthermore, cats that were identified as more anxious around humans upon arrival at the shelter had a tendency to spend more time producing slow blink sequences.

Evidence indicates that displays typically seen in both positive and submissive contexts often share facial muscle movements [26]. For example, the human smile and the silent bared teeth display in non-human primates can be seen across a range of contexts, including affiliative and conflict reduction situations [27,28,29]. The results here suggest a potentially analogous dual function for the slow blink sequence in domestic cats. These expressions in humans and non-human primates can help individuals to de-escalate negative social interactions as well as promote positive ones [28,29,30]. In these instances, positive communicative displays can serve a generalized purpose of enhancing social affinity between partners. Slow blinking could share a similar social bonding function, and therefore the trend towards an increased length of time spent slow blinking seen in the anxious cats in our study may have been used to mitigate cats’ anxiety around humans. This could also explain the presence of half blinking in fearful contexts around humans in another feline facial behaviour study [31]. Such down-regulation in social contexts could also be considered a form of submissive behaviour. Thus, positive signals may have derived from submissive displays and become more complex as social complexity increased, continuing to serve a dual function in this respect. Research on the similarities between positive and submissive displays is an important line of study. Future research could usefully consider slow blinking in cats in this context, with a wider sample of anxious and non-anxious cats.

Similar to the results in our study, there is evidence that particular facial actions (inner brow raiser AU101) in dogs can increase the speed of their adoption in shelters [23]. While it was suggested that this display may operate through enhancing paedomorphic facial features in dogs, it was also noted that the inner brow raise action may have been perceived as indicating sadness (the corresponding action in humans (AU 1) is an integral feature in typical human sadness expressions). In the current study, eye closure movements in cats increased adoption speed. Narrowing of the eye aperture shares similar features with the human Duchenne smile—the genuine smile in humans [32]. This is interesting as humans not only use the eyes to gauge the emotional state of others [33], but also to gain purposeful social information [34]. Thus, the adopters may have responded more to cats who made eye closures as they appeared happier, and potentially friendlier to prospective adopters.

The apparent response to cat eye closures by adopters, rather than other eye narrowing movements in this study (half blinking and eye narrowing), might be the result of eye closures lasting longer than the other eye narrowing movements (see Table 1). It is possible then that the salience of eye closures may affect potential adopters more than other eye narrowing movements. This is supported by the human literature that shows that the eyes play an important role in influencing human behaviour in a number of contexts [33,34,35]. For example, eyes that are made visually explicit can enhance the likelihood of altruistic behaviour in humans [35]. Humans may therefore be inadvertently influenced by eye closures but not other, more subtle, eye narrowing movements. Interestingly, however, cats do not appear to use eye closures more than other eye narrowing movements in their slow blink sequences. This suggests that eye closures, specifically, may not have undergone selective pressure by humans but the overall dynamic pattern of the slow blink sequence may have. However, since eye closure is not the only, or the most prevalent, AU in the slow blink sequence, and our sample size is limited, these results are tentative and future research should confirm the findings.

In another published study, none of the cat facial actions described in CatFACS were related to adoption rates in a shelter environment [24], but cats’ rubbing behaviour was related to faster speeds of rehoming. Interestingly, the authors also found that rubbing was correlated with half blinking and blinking in an exploratory factor analysis. Reference [24] study may not have provided sufficient opportunity for cats to display slow blinking behaviour as the human-cat paradigm used was non-communicative in nature. The social aspect of slow blinking may therefore explain the influence of eye closures on potential adopters. Adopters from previous studies, when asked the reasons for choosing their pet, often highlight the connection they felt towards the individual, e.g., “we clicked” and “the cat chose us” [36]. Since the slow blink is becoming increasingly recognised as a form of communication employed by cat owners and non-cat owners alike, these findings may have practical implications for shelters by introducing strategies to promote positive social interactions between potential adopters and shelter cats, particularly for cats that might be more likely to spend a longer time in care (e.g., inactive cats, [37]; or black cats, [38]). Alternatively, other cat characteristics, such as age and breed, may modulate the relationship between eye closure and time to rehome. A larger sample size would be required to support the multivariate analyses necessary to investigate this further and would be an interesting area for future research.

## 5. Conclusions

Our study shows that shelter cats participate in slow blinking interactions with humans, and that this interaction may be linked to faster rehoming rates for shelter cats. Additionally, we demonstrate a trend that suggests that nervous cats spend more time slow blinking, providing supporting evidence that this behaviour may act as both a positive signal and a submissive display. Increased knowledge about feline behaviour acts as a protective factor against the relinquishment of cats [39]. Thus, better understanding of human-cat communication, such as the slow blink, is fundamental to the welfare of cats. Future studies should further explore the function of slow blinking in cats in a variety of contexts. Further research could also examine how the use of slow blinking may enhance cat-human attachment.

## Figures and Tables

**Table 1:** Cat and human eye movements and corresponding facial action units (AU; Facial Action Coding System). See [24,25] for descriptions and photographs of the actions described.

Code Name	Facial Action Unit	Description of Code
Cat Half Blink	AU 147	One of the eyelids (upper or lower) moves towards the other without ever closing the eye. It can occur in only one eye. It may occur in a succession of movements or one movement only.
Cat Eye Closure	AU 143	The upper and lower eyelids move towards each other and cover the eye completely. The eye has to remain closed for more than half a second. It can occur in only one eye.
Cat Eye Narrowing		The upper and lower eyelids are held half closed. This is a prolonged version of AU147.
Cat Eye Closures due to Movement		When a cat closes its eyes due to rubbing against a surface, scratching, yawning or any other movement that would naturally cause the eyes to narrow or close.
Human Eye Closure	AU 43	The upper and lower eyelids move towards each other and cover the eye completely. The eye has to remain closed for more than half a second.
Human Eye Narrowing		The upper and lower eyelids are held half closed. The eye aperture is held partially closed for at least 2 frames, as in Cat Eye Narrowing.

## Supplementary Materials

The following are available online at https://www.mdpi.com/2076-2615/10/12/2256/s1, Table S1: Full dataset.

## Appendix A

No significant difference was found between desensitisation and non-desensitisation groups in the duration (desensitisation: M = 2.39 ± 2.36; non-desensitisation: M = 2.93 ± 3.26; U = 33.00, *p* = 0.57) or number (desensitisation: M = 3.00 ± 2.98; non-desensitisation: M = 5.20 ± 4.47; U = 28.00, *p* = 0.32) of half blinks. There were no significant differences between the desensitisation and non-desensitisation groups in the duration (desensitisation: M = 12.78 ± 14.14; non-desensitisation: M = 8.82 ± 9.72; U = 31.00, *p* = 0.46) or number (desensitisation: M = 4.00 ± 2.88; non-desensitisation: M = 2.90 ± 2.08; U = 30.50, *p* = 0.41) of eye narrowing. The desensitisation groups also did not significantly differ in the duration (desensitisation: M = 16.73 ± 22.73; non-desensitisation: M = 6.24 ± 10.83; U = 30.00, *p* = 0.41) or number (desensitisation: M = 1.63 ± 1.92; non-desensitisation: M = 1.00 ± 1.41; U = 31.00, *p* = 0.46) of eye closures. There was no significant difference in the average response latency of desensitisation and non-desensitisation cats (desensitisation: M = 6.12 ± 2.01; non-desensitisation: M = 7.22 ± 1.78; U = 29.00, *p* = 0.33).
